# Reflective chiral meta-holography: multiplexing holograms for circularly polarized waves

**DOI:** 10.1038/s41377-018-0019-8

**Published:** 2018-06-27

**Authors:** Qiu Wang, Eric Plum, Quanlong Yang, Xueqian Zhang, Quan Xu, Yuehong Xu, Jiaguang Han, Weili Zhang

**Affiliations:** 1 Center for Terahertz Waves and College of Precision Instrument and Optoelectronics Engineering, Tianjin University and the Key Laboratory of Optoelectronics Information and Technology (Ministry of Education), Tianjin, 300072 China; 2 Optoelectronics Research Centre and Centre for Photonic Metamaterials, University of Southampton, Highfield, Southampton, SO17 1BJ UK; 3 School of Electrical and Computer Engineering, Oklahoma State University, Stillwater, OK 74078 USA

## Abstract

By allowing almost arbitrary distributions of amplitude and phase of electromagnetic waves to be generated by a layer of sub-wavelength-size unit cells, metasurfaces have given rise to the field of meta-holography. However, holography with circularly polarized waves remains complicated as the achiral building blocks of existing meta-holograms inevitably contribute to holographic images generated by both left-handed and right-handed waves. Here we demonstrate how planar chirality enables the fully independent realization of high-efficiency meta-holograms for one circular polarization or the other. Such circular-polarization-selective meta-holograms are based on chiral building blocks that reflect either left-handed or right-handed circularly polarized waves with an orientation-dependent phase. Using terahertz waves, we experimentally demonstrate that this allows the straightforward design of reflective phase meta-holograms, where the use of alternating structures of opposite handedness yields independent holographic images for circularly polarized waves of opposite handedness with negligible polarization cross-talk.

## Holography: Lighting the path to new applications for holographic imaging

A new technique for creating holograms from left- or right-handed circularly polarized light could enable new applications in communications, data storage and three-dimensional displays. Holography provides a promising way to design and reconstruct high-quality, three-dimensional images using light. However, spatial light modulators used to create holograms control only either the intensity or phase of light, have limited spatial resolution and cannot control left- or right-handed circularly polarized light independently. This led a team of researchers led by Jiaguang Han from Tianjin University and Eric Plum from University of Southampton to use chiral metasurfaces to control left- and right-handed electromagnetic waves independently with much higher spatial resolution. The work has shown how to combine different functionalities for left- and right-handed polarized light into a single device, and could lead to new holographic imaging applications.

## Introduction

Holography, a three-dimensional (3D) imaging technique, was originally proposed by Gabor^1^. Initial holograms recorded interference fringes of an object beam and a reference beam to store both the phase and amplitude information of the object. When such a hologram is illuminated by the same reference beam, a 3D image of the object will be reconstructed at the object’s original position. Rapid advances of computer science and optoelectronics in 1960s led to computer-generated holography^2^. Hologram generation by numerical calculations eliminates the need for real objects, making computer-generated holography more widely applicable. This design approach can be applied across the whole-electromagnetic spectrum and even in acoustics^3^. By employing spatial light modulators (SLMs) as holograms, high-quality holographic imaging and dynamic holographic displays have been demonstrated. However, spatial light modulators can only control either the intensity or phase of electromagnetic waves with a limited spatial resolution due to “pixels,” which are large compared to the wavelength. In contrast, metasurfaces allow simultaneous control over the amplitude and phase of electromagnetic waves with a much higher spatial resolution due to sub-wavelength pixelation^4–11^, which allows unwanted conjugate images (that occur in conventional holography) to be eliminated in metasurface holography (meta-holography). Meta-holography has become a significant research direction that is applicable from 2D imaging to multi-plane imaging^12–14^ and 3D imaging^15^, and it offers great potential for communication, data storage, beam shaping, and 3D displays. Benefiting from the rapid development and unique characteristics of metasurfaces, high-efficiency meta-holography, broadband meta-holography, and actively tunable meta-holography have been demonstrated by employing reflection-type^16,17^ or dielectric^18^ metasurfaces, geometric metasurfaces^13–16,18^, and stretchable metasurfaces^19^, respectively. Additionally, the ability to engineer the polarization, spectral and nonlinear properties of metasurface unit cells has enabled polarization-controlled holography^20–24^, multi-color holography^25–28^, and nonlinear holography^29,30^. However, independent generation of different holographic images for circularly polarized waves of opposite handedness remains challenging since the achiral resonators used in existing approaches inevitably contribute to both holographic images.

**Fig. 1:**
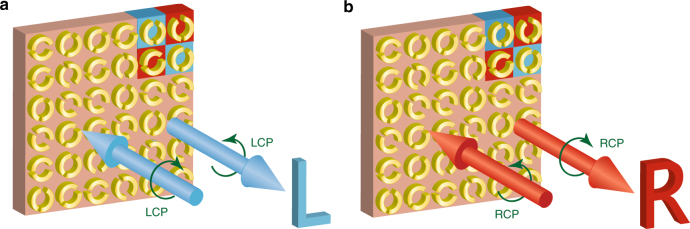
Reflective chiral meta-holography. Images (**a**) “L” and (**b**) “R” are reconstructed at the same position for illumination with circularly polarized waves of opposite handedness. Blue cells only reflect LCP, and red cells only reflect RCP, as shown by Fig. 2. The meta-hologram has the unusual property that it does not reverse the handedness of circularly polarized waves upon reflection

Here we show how planar chirality enables the creation of meta-holograms for one circular polarization or the other. By alternating chiral resonators that only contribute to holographic images for either left-handed or right-handed waves, we demonstrate a straightforward method for multiplexing reflective holograms for circularly polarized waves. We experimentally show that the resulting meta-hologram generates independent holographic images for reflected terahertz waves of opposite handedness. In contrast to conventional reflectors, the planar chiral meta-hologram does not change the handedness of circularly polarized waves upon reflection (Fig. 1).

## Materials and methods

### Meta-hologram sample fabrication

**Fig. 2:**
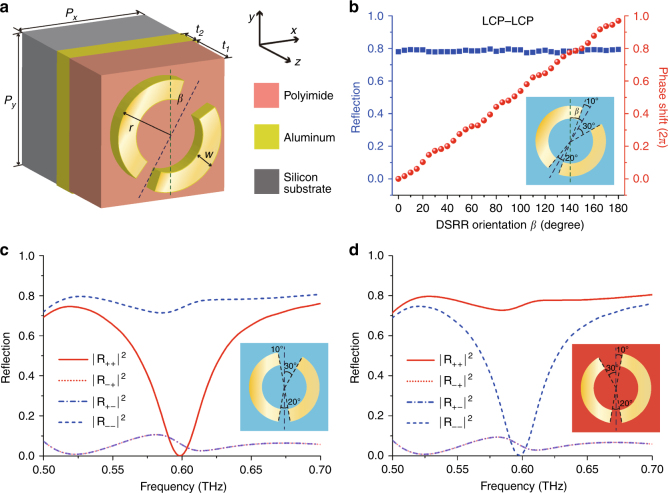
Planar chiral unit cells and their simulated reflection characteristics. **a** Schematic of a double-split ring resonator (DSRR) patterned on a three-layer structure. *r*, *w*, and *β* represent the outer radius, line width, and orientation angle of the DSRR, respectively. *P*_*x*_ and *P*_*y*_ are the periods of the metasurface lattice, and *t*_1_ and *t*_2_ represent the thicknesses of the polyimide layer and the aluminum layer, respectively. **b** The intensity |E|^2^ and phase shift of the LCP component of the reflected electric field for LCP illumination of the L-type DSRR array at 0.6 THz. Reflectivity spectra of the **c** L-type and **d** R-type DSRR arrays for orientation *β* = 0 in terms of circularly polarized intensities. |*R*_+__−_|^2^ represents the fraction of incident LCP (−) that will be reflected as RCP (+)

The meta-hologram consists of 100 × 100 aluminum double-split ring resonators (DSRRs) that are 200 nm thick on a 43 μm thick polyimide layer backed by a 200 nm thick aluminum mirror and supported by a 500 μm thick silicon wafer. The DSRRs are arranged in a lattice with periods *P*_*x*_ =*P*_*y*_ = 170 μm and they have an outer radius *r* = 68 μm, line width *w* = 25 μm and orientation *β* (Fig. 2a).

The reflective chiral meta-hologram was fabricated using conventional photolithography. Starting with a 500 μm thick silicon wafer substrate, a 200 nm thick-Al layer was deposited using thermal evaporation. Then, a 43 μm thick layer of polyimide was spin-coated. Next, a layer of photoresist (AZ P4000) was spin-coated on the polyimide layer, and the DSRR patterns were exposed using conventional photolithography. After development, a 200 nm thick layer of Al was deposited on the sample to form the DSRRs. Finally, the remaining photoresist and Al outside the DSRRs were removed using a lift-off process.

### Electromagnetic simulations of the chiral unit cells

Modeling of the electromagnetic properties of the DSRRs was performed using CST Microwave Studio (CST Computer Simulation Technology GmbH, Darmstadt, Germany) based on the above DSRR dimensions, describing aluminum with a conductivity of 3.72 × 10^7^ S·m^−1^ and polyimide with a permittivity of *ε* = 2.93 + 0.13i. Periodic boundary conditions were applied in both the *x* and *y* directions, while the perfectly matched-layer (PML) boundary condition was applied in the *z* direction. Normally incident *x*-polarized and *y*-polarized plane waves were used to excite the DSRR structures, and a probe was set before the structure to detect both the *x*-polarized and *y*-polarized components of the reflected electric field. Using of a time-domain solver, we removed the incident signal in the time-domain first and then calculated the reflected electric field in the frequency domain using a Fourier transform. Thus, the simulated reflection coefficients for linearly polarized waves were obtained, that is, *R*_*xx*_, *R*_*yy*_, *R*_*xy*_, and *R*_*yx*_. The reflection coefficients for circularly polarized waves were then calculated using the following:

1 $$\left( {\begin{array}{*{20}{c}} {R_{ + + }} \quad {R_{ + - }} \\ {R_{ - + }} \quad {R_{ - - }} \end{array}} \right)\, \hskip13pc \\ \hskip-1.0pc= \frac{1}{2}\left( {\begin{array}{*{20}{c}} {R_{xx} - R_{yy} + i(R_{xy} + R_{yx})} \quad {R_{xx} + R_{yy} - i(R_{xy} - R_{yx})} \\ {R_{xx} + R_{yy} + i(R_{xy} - R_{yx})} \quad {R_{xx} - R_{yy} - i(R_{xy} + R_{yx})} \end{array}} \right)$$

where + and − refer to right-handed circular polarization (RCP) and left-handed circular polarization (LCP), respectively, and *R*_*ij*_ represents the *i*-polarized reflected electric field component in response to a *j*-polarized incident electric field of amplitude 1, $i,j \in \,\{ x,y, + , - \}$. Here, RCP is defined as a clockwise rotation of the electric field vector at a fixed point when looking into the beam.

### Reflective chiral meta-hologram design procedure

**Fig. 3:**
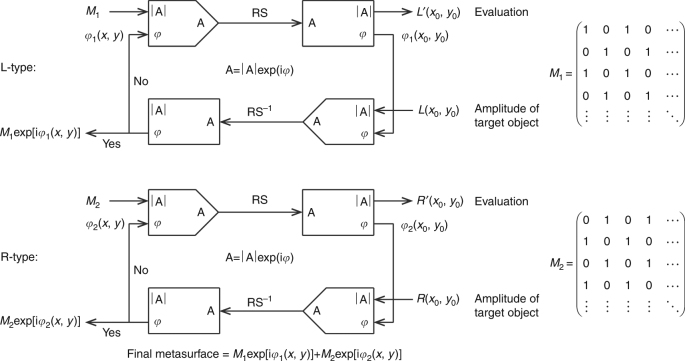
Meta-hologram design based on the partitioned iterative algorithm. The initial input phase distributions are random for both flow charts. RS and RS^−1^ represent the Rayleigh–Sommerfeld diffraction formula and the “inverse” Rayleigh–Sommerfeld diffraction formula, respectively. |A| and *φ* represent the amplitude and phase distributions, respectively

To design the reflective chiral phase-only meta-hologram, a partitioned iterative algorithm was applied to obtain the desired electric field phase distribution in the plane of the metasurface structure, see Fig. 3. There are many phase retrieval algorithms, including the Gerchberg–Saxton^31^, Fienup Fourier^32,33^, and Yang–Gu^34^ algorithms. For our partitioned iterative algorithm, we chose the conventional Gerchberg–Saxton algorithm because it is simple, widely applied, and able to produce high-quality images, as discussed below. The desired distributions of the two types of DSRRs are optimized separately and then combined to compose the final metasurface. The initial input phase distributions are random for both flow charts. As our metasurface is 17 mm wide (along both the *x* and *y* directions) and uses an imaging distance of *z* = 35 mm, it does not satisfy the Fresnel approximation in diffraction optics, namely, $z \gg \,\sqrt {(x - x_0)^2\, + \,(y\, - \,y_0)^2}$, where (*x*_0_, *y*_0_) is a fixed point in the image plane and (*x*, *y*) is an arbitrary point on the metasurface. Thus, the Fresnel diffraction formula that is usually used in the conventional Gerchberg–Saxton algorithm is not sufficiently accurate and is replaced by the Rayleigh–Sommerfeld diffraction formula

2 $$U(x_0,y_0)\, = \,\frac{1}{{i\lambda }}{\iint} {U(x,y)\cos \, < {\mathbf{n}},{\mathbf{r}} > \,\frac{{\exp (ikr)}}{r}{\rm{d}}x{\rm{d}}y}$$

which corresponds to “RS” in Fig. 3. Here, *U*(*x*_0_, *y*_0_) and *U*(*x*, *y*) represent the electric fields on the image plane and metasurface, respectively; *λ* is the wavelength in vacuum; $r\, = \,\sqrt {(x_0 - x)^2 + (y_0 - y)^2}$; and $\cos \, < \,{\mathbf{n}},{\mathbf{r}} > = z{\mathrm{/}}r$ is the inclination factor. The amplitude of the reconstructed image is then evaluated. Take the first flow chart as an example; if the reconstructed image is evaluated to be not good enough, then by combining the amplitude distribution of the target object *L*(*x*_0_, *y*_0_) with the calculated phase distribution *φ*_1_(*x*_0_, *y*_0_), the new electric field, *U’*(*x*_0_, *y*_0_) = *L*(*x*_0_, *y*_0_) ∙ exp[i*φ*_1_(*x*_0_, *y*_0_)], becomes the input and is applied in the “inverse” process with the Equations 3 and 4: *U’* and *L’* are set correctly in the pdf proof, but the prime is too low and too large in the eProofing environment.

3 $$U\prime (x,y)\, = \frac{1}{{i\lambda }}{\iint} {U\prime (x_0,y_0)\cos < {\mathbf{n}},{\mathbf{r}} > \frac{{\exp ( - ikr)}}{r}{\rm{d}}x_0{\rm{d}}y_0}$$

which corresponds to “RS^−1^” in Fig. 3. By combining the amplitude distribution *M*_1_ with the new calculated phase distribution, the circulation proceeds. The iteration will not terminate until the reconstructed image quality meets the requirement

4 $${\iint} {\left| {L\prime (x_0,y_0)^2 - \,L(x_0,y_0)^2} \right|{\rm{d}}x_0{\rm{d}}y_0} < \varepsilon$$

where *ε* is a number. *M*_1_ and *M*_2_ are two complementary “masks” corresponding to the distributions of L-type and R-type DSRRs in the metasurface, respectively, which can be seen in Fig. 3. Once the two iteration processes are complete, the metasurface composed of the two types of DSRRs can be determined.

### Experimental hologram characterization

**Fig. 4:**
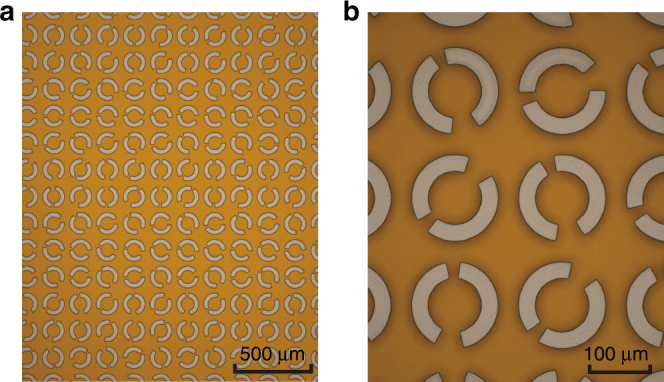
The fabricated meta-hologram. **a** Partial optical microscope image of the reflective chiral meta-hologram. **b** Magnified section of the meta-hologram

**Fig. 5:**
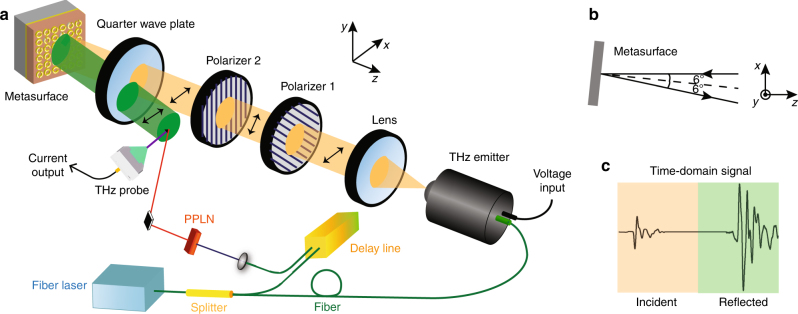
Measurement setup. **a** Schematic diagram of the reflective fiber-based near-field scanning terahertz microscopy setup. PPLN represents a lithium niobate crystal used for frequency doubling. A small angle of incidence on the metasurface spatially separates the incident wave and reflected field, allowing holographic images to be read by the THz probe. **b** Top view of the region near the metasurface in **a**. **c** Schematic of the detected terahertz time-domain signal. The yellow and green regions represent the incident and reflected time-domain components, respectively

The meta-hologram (Fig. 4) was characterized using reflective fiber-based near-field scanning terahertz microscopy, which is schematically illustrated in Fig. 5a. Fiber laser pulses with an ~50 fs pulse width and 1550 nm central wavelength were split into two beams that were used to generate the terahertz radiation and to detect the reflected terahertz waves, respectively. The terahertz wave was first emitted by a commercial photoconductive antenna and then collimated by a TPX terahertz lens. Two metallic grid polarizers were placed after the lens. The first polarizer was placed with a 45° orientation with respect to the *x*-axis, and the second was placed along the *y*-axis or the *x*-axis to produce *x*-polarized or *y*-polarized terahertz waves. A quarter wave plate working at 0.6 THz was located before the metasurface to transform the incident linearly polarized states into circularly polarized states. LCP and RCP were selected for illumination of the reflective metasurface by a 90° rotation of the quarter wave plate. After reflection, the outgoing LCP or RCP were transformed into linearly polarized waves by passing through the quarter wave plate a second time and then were detected by a commercial terahertz near-field probe. The photoconductive antenna gap of the probe was set along the *x*-axis to detect only the *x*-polarized component of the reflected electric field. When the second polarizer was oriented to transmit *x*-polarized waves that were transformed into LCP by the quarter wave plate, the detection corresponded to the result of the LCP–LCP channel. Then, the measurement of the RCP–RCP channel was achieved by simply rotating the quarter wave plate by 90°. To experimentally detect the electric field distributions of the polarization conversion channels, namely, LCP–RCP, and RCP–LCP, the second polarizer was rotated by 90° to transmit *y*-polarized waves, resulting in a reversal of the handedness of the circularly polarized waves illuminating the metasurface. Here, LCP–RCP corresponds to incident LCP and detected RCP intensities. Note that the probe is fixed to detect only the *x*-polarized electric field component during the whole-experimental process.

To prevent the probe from blocking the incident wave, the metasurface was placed with a 6° inclination to spatially separate the incident wave and reflected wave in the detection region, as shown in Fig. 5b. In fact, the detected time-domain signal also contains a small incident component, as shown in Fig. 5c. However, there is enough time delay between the incident pulse and reflected pulse to cut the incident component off in the time domain and then obtain the frequency response at 0.6 THz from the reflected pulse using a Fourier transform. These results also indicate that the probe slightly shielded the incident wave. However, this shielding could be ignored as the probe was sufficiently small and far from the optical axis.

## Results and discussions

The essential building block of a reflective meta-hologram for only one circular polarization is a unit cell that reflects waves of one circular polarization with a high efficiency while absorbing waves of the other circular polarization. If no static magnetic field is present, and thus no Faraday effect, such handedness-selective properties require chirality. Conventional 3D-chiral structures (e.g., helical wires) exhibit circular dichroism, resulting in different absorption levels for LCP and RCP, which could be the basis of a circular-polarization-selective meta-hologram operating in transmission. However, for a reflecting structure, the handedness reversal of the wave upon reflection would undo any polarization contrast during the second pass through the 3D-chiral structure. Instead, circular-polarization-selectivity for reflected waves is achieved by a 2D-chiral (planar chiral) structure (e.g., a flat spiral), which has the opposite handedness for observation (or illumination) from opposite sides. A single 2D-chiral layer may exhibit up to 50% larger absorption for one circular polarization than for the other^35,36^, and when combined with a mirror, one circular polarization can, in principle, be fully absorbed while the other is fully reflected without a handedness change^37^. We use two types of 2D-chiral double-split ring resonators (DSRRs) that are backed by a mirror as the unit cells of our meta-hologram to achieve circular-polarization-selective reflection with a high efficiency, see Fig. 2a and Methods section. A lossy polyimide layer between the aluminum DSRRs of sub-wavelength size and the aluminum mirror allows the absorption of terahertz waves. The DSRRs themselves consist of two segments of a circle of different lengths (140°, 160°) separated by gaps of different sizes (20°, 40°), resulting in a planar structure that has mirror-asymmetry and therefore 2D chirality. The DSRR orientation *β* is defined such that the lower split of 20° is symmetric with respect to the *y*-axis for *β* = 0°. For this orientation, we call the DSRR “L-type” when 30° of the upper split are in the first quadrant (as shown in Fig. 2b, c), and “R-type” when 30° of the upper split are in the second quadrant (as shown in Fig. 2d). The L-type and R-type DSRRs are mirror images of each other.

We modeled and optimized the unit cell dimensions using CST Microwave Studio as explained in Methods section. Our simulations show that, for the L-type DSRR, the intensity conversion from LCP to LCP (|*R*_*−−*_|^2^) is very high and remains at an approximately constant value of 0.8 at 0.6 THz when the L-type DSRR is rotated around the *z*-axis, as shown in Fig. 2b. As the DSRR orientation *β* increases from 0 to π, the phase of the reflected LCP component gradually changes from 0 to 2π and approximately follows a 2*β* dependence, which is the expected behavior for such a geometric phase, also known as the Pancharatnam–Berry phase. While |*R*_*−−*_|^2^ is large, all of the other reflectivities are small, at 0.6 THz, see Fig. 2c. Similarly, a large polarization contrast has been experimentally observed for uniform arrays of ~100× larger chiral DSRR structures operating at microwave frequencies^37^. This phenomenon results from the different electromagnetic responses of the chiral DSRR to illumination with circularly polarized waves of opposite handedness. Incident LCP excites a low-loss electric dipole resonance that scatters as LCP, while incident RCP excites a magnetic dipole resonance that traps electromagnetic energy until it is absorbed by the lossy polyimide layer^36^. Due to their symmetry relationship, the L-type DSRR (Fig. 2c) and R-type DSRR (Fig. 2d) have interchanged reflectivities for LCP and RCP. The phase of the reflected LCP can be controlled by rotating the L-type DSRR (Fig. 2b), and the phase of the reflected RCP can similarly be controlled by rotating the R-type DSRR without changing the reflected intensity. Thus, L-type and R-type DSRRs allow the realization of high-efficiency reflective phase meta-holograms for LCP and RCP. Such circular-polarization-selective meta-holograms will reflect circularly polarized waves without a handedness change and with negligible polarization cross-talk.

Here we alternate L-type and R-type DSRRs to design a reflective chiral hologram that projects holographic images of the letters “L” and “R” in the same location for illumination by LCP and RCP, respectively, as shown in Fig. 1. The two types of DSRRs alternate in both the *x* and *y* directions to ensure that the reconstructed image has the same spatial resolution along the two directions, which can be seen in the top right corner of the metasurface in Fig. 1. The image plane is designed to be 35 mm away from the metasurface. The whole metasurface is a 100 × 100 grid of unit cells with an overall size of 17 × 17 mm.

The reflective chiral meta-hologram was designed using a partitioned iterative algorithm to obtain the desired electric field phase distribution in the meta-hologram plane, as explained in Methods section and illustrated in Fig. 3. The optimized phase distribution of the LCP hologram determines the orientation *β* of each L-type DSRR, while the phase distribution of the RCP hologram determines the orientation of each R-type DSRR. The reflective chiral meta-hologram was then fabricated using conventional photolithography, as explained in Methods section. Figure 4 shows part of the fabricated chiral meta-hologram.

**Fig. 6:**
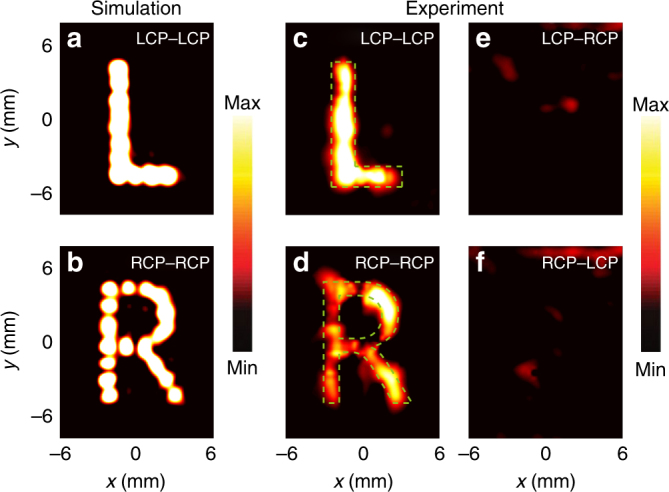
Multiplexed holographic images read with circularly polarized waves. **a**, **b** Simulated and **c**, **d** measured LCP and RCP intensity distributions (|E|^2^) on the image plane at 0.6 THz, respectively. The green dashed lines represent the target image profiles for “L” and “R”. **e**, **f** Intensity conversion between opposite circular polarizations measured on the image plane at 0.6 THz. LCP–RCP corresponds to incident LCP and detected RCP intensities

Figure 6a, b show the calculated holographic images of “L” and “R” on the image plane based on the optimized phase distributions of the hologram as determined by the iterative processes. These simulated results agree well with the design, including the image sizes, line widths, and relative intensity distributions. Considering the metasurface size (17 × 17 mm), imaging distance (35 mm), and wavelength (500 μm), the diffraction limit for the image plane resolution is 664 μm according to the Rayleigh criterion. As the resolution of the simulated images is close to this limit, the use of more advanced phase retrieval algorithms would not yield substantial improvements.

The holographic images generated by the meta-hologram were experimentally characterized using reflective fiber-based near-field scanning terahertz microscopy, as explained in Methods section and illustrated in Fig. 5. Figure 6c–f shows the measured holographic images on the image plane for all possible combinations of incident and detected circularly polarized waves at 0.6 THz. All of the electric field distributions are detected in the image plane (*z* = 35 mm) at 0.3 mm intervals from −6 to 6 mm in the *x* direction and from −7.5 to 7.5 mm in the *y* direction. The measured LCP and RCP holographic images shown in Fig. 6c, d are in agreement with the simulations, including the location, size, and profile of the images. Circular polarization conversion is negligible (Fig. 6e, f), as expected. Cross-talk between the images is very small and was not detected between the main LCP and RCP holographic images. Compared with the simulations, there is a small decline in the experimental image quality, which mainly results from fabrication imperfections and the complexity of the experimental setup, where the metasurface is placed at a 6° inclination to separate the incident and reflected waves to prevent the probe from blocking the incident wave. Thus, the incident and detected terahertz waves do not strictly propagate normal to the metasurface. With regard to the fabrication process, there are inevitable imperfections in the flatness and thickness of the polyimide layer, which influences the electromagnetic responses of the DSRRs.

The measured holographic imaging efficiency, defined as the ratio between the measured terahertz power in the imaging plane’s “L” or “R” regions (green dashed profiles in Fig. 6c, d) and the input power at 0.6 THz, reaches 35% and 19% for the LCP and RCP images, respectively. As seen in Fig. 6d, part of the intensity in the measured “R” image is outside the designed “R” region, which is the main cause of the lower holographic imaging efficiency of the RCP image. Indeed, the window efficiency, defined as the ratio between the detected terahertz power on the whole image plane (12 × 15 mm) and the input power at 0.6 THz, is 39% and 32% for the LCP and RCP images, respectively, showing a much smaller difference. To obtain the measured holographic imaging and window efficiencies, the input power was measured by detecting the reflected power in the same image plane after replacing the metasurface with a gold mirror. As the gold mirror converts the incident LCP completely into reflected RCP, we choose the LCP–RCP channel as the reference.

We argue that our approach to multiplexing holograms for circularly polarized electromagnetic waves is applicable to any spectral range, from microwaves to visible light, by adjusting the overall size of the chiral unit cells. Indeed, non-holographic structures with ~100× larger unit cells have already been shown to yield a large polarization contrast for microwaves^37^, and high-efficiency meta-holograms for visible light consisting of achiral nanostructures backed by a mirror have been reported^16,17,22,23,27^. In contrast to broadband spatial light modulators, which are commonly used for dynamic generation of holograms with visible light, static chiral meta-holograms, as reported here, offer spectral selectivity and circular polarization selectivity, as well as sub-wavelength pixelation.

The meta-hologram’s polarization-selective functionality arises from its chiral building blocks that support an absorption resonance for either RCP or LCP, resulting in a fractional bandwidth of a few percent for our structure (Fig. 2c). However, there are several strategies that could lead to more broadband, as well as spectrally tunable chiral meta-holograms. The bandwidth could be increased by broadening the resonance, for example, by replacing polyimide with a more lossy material, by increasing the asymmetry of the chiral split rings or by combining chiral elements with different resonance frequencies^38^. Spectrally tunable meta-holograms could be realized by microelectromechanical control of the metasurface-to-mirror spacing^39^ or by replacing polyimide with a phase change material^40^.

## Conclusion

We demonstrate a straightforward solution for circular-polarization-selective meta-holography. Using planar chiral unit cells, we realize reflective phase meta-holograms that produce an image when illuminated by waves of one circular polarization while absorbing waves of the other circular polarization. By alternating planar chiral elements of opposite handedness, we generate a meta-hologram with fully independent images that are revealed by illumination with circularly polarized waves of the opposite handedness. Such reflective holograms do not change the handedness of circularly polarized waves upon reflection and show a high efficiency and negligible polarization cross-talk. As the phases of the LCP and RCP reflected waves are controlled independently by the orientation of LCP and RCP resonators (Pancharatnam–Berry phase), the design of such multiplexed meta-holograms is straightforward and simple. More generally, we demonstrate how completely independent optical functionalities for left and right circularly polarized waves can be combined in a single device, which can be applied in reflective holographic imaging and may lead to dual-channel holographic displays, e.g., when implemented based on reconfigurable metasurfaces^41,42^. Beyond holographic imaging, our approach enables selective and multiplexed metasurface devices for circularly polarized waves, e.g., devices that redirect or focus waves of opposite handedness independently for applications, such as polarimetry, polarization-selective image detection, and spatial separation of polarization-encoded information channels.

## References

[1] Gabor D (1948). A new microscopic principle. Nature.

[2] Lohmann AW, Paris DP (1967). Binary Fraunhofer holograms, generated by computer. Appl. Opt..

[3] Melde K, Mark AG, Qiu T, Fischer P (2016). Holograms for acoustics. Nature.

[4] Yu NF (2011). Light propagation with phase discontinuities: generalized laws of reflection and refraction. Science.

[5] Kildishev AV, Boltasseva A, Shalaev VM (2013). Planar photonics with metasurfaces. Science.

[6] Meinzer N, Barnes WL, Hooper IR (2014). Plasmonic meta-atoms and metasurfaces. Nat. Photonics.

[7] Lin DM, Fan PY, Hasman E, Brongersma ML (2014). Dielectric gradient metasurface optical elements. Science.

[8] Wang Q (2016). Optically reconfigurable metasurfaces and photonic devices based on phase change materials. Nat. Photonics.

[9] Chen WT (2017). Generation of wavelength-independent subwavelength Bessel beams using metasurfaces. Light Sci. Appl..

[10] Wang Q (2016). Broadband metasurface holograms: toward complete phase and amplitude engineering. Sci. Rep..

[11] Ni XJ, Kildishev AV, Shalaev VM (2013). Metasurface holograms for visible light. Nat. Commun..

[12] Wei QS, Huang LL, Li XW, Liu J, Wang YT (2017). Broadband multiplane holography based on plasmonic metasurface. Adv. Opt. Mater..

[13] Huang K (2016). Silicon multi-meta-holograms for the broadband visible light. Laser Photonics Rev..

[14] Huang LL (2015). Broadband hybrid holographic multiplexing with geometric metasurfaces. Adv. Mater..

[15] Huang LL (2013). Three-dimensional optical holography using a plasmonic metasurface. Nat. Commun..

[16] Zheng GX (2015). Metasurface holograms reaching 80% efficiency. Nat. Nanotechnol..

[17] Yifat Y (2014). Highly efficient and broadband wide-angle holography using patch-dipole nanoantenna reflectarrays. Nano Lett..

[18] Devlin RC, Khorasaninejad M, Chen WT, Oh J, Capasso F (2016). Broadband high-efficiency dielectric metasurfaces for the visible spectrum. Proc. Natl Acad. Sci. USA.

[19] Malek SC, Ee HS, Agarwal R (2017). Strain multiplexed metasurface holograms on a stretchable substrate. Nano Lett..

[20] Xie ZW (2017). Meta-holograms with full parameter control of wavefront over a 1000 nm bandwidth. ACS Photonics.

[21] Mueller JPB, Rubin NA, Devlin RC, Groever B, Capasso F (2017). Metasurface polarization optics: independent phase control of arbitrary orthogonal states of polarization. Phys. Rev. Lett..

[22] Wen DD (2015). Helicity multiplexed broadband metasurface holograms. Nat. Commun..

[23] Chen WT (2014). High-efficiency broadband meta-hologram with polarization-controlled dual images. Nano Lett..

[24] Wang Q (2017). Polarization and frequency multiplexed terahertz meta-holography. Adv. Opt. Mater..

[25] Li X (2016). Multicolor 3D meta-holography by broadband plasmonic modulation. Sci. Adv..

[26] Wan WW, Gao J, Yang XD (2016). Full-color plasmonic metasurface holograms. ACS Nano.

[27] Huang YW (2015). Aluminum plasmonic multicolor meta-hologram. Nano Lett..

[28] Walther B (2012). Spatial and spectral light shaping with metamaterials. Adv. Mater..

[29] Ye WM (2016). Spin and wavelength multiplexed nonlinear metasurface holography. Nat. Commun..

[30] Almeida E, Bitton O, Prior Y (2016). Nonlinear metamaterials for holography. Nat. Commun..

[31] Gerchberg RW, Saxton WO (1972). A practical algorithm for the determination of phase from image and diffraction plane pictures. Optik.

[32] Fienup JR (1978). Reconstruction of an object from the modulus of its Fourier transform. Opt. Lett..

[33] Fienup JR (1980). Iterative method applied to image reconstruction and to computer-generated holograms. Opt. Eng..

[34] Yang GZ, Gu BY (1981). On the amplitude-phase retrieval problem in the optical systems. Acta Phys. Sin..

[35] Plum E (2010). Chirality and Metamaterials.

[36] Plum E, Fedotov VA, Zheludev NI (2009). Planar metamaterial with transmission and reflection that depend on the direction of incidence. Appl. Phys. Lett..

[37] Plum E, Zheludev NI (2015). Chiral mirrors. Appl. Phys. Lett..

[38] Liu YH, Gu S, Luo CR, Zhao XP (2012). Ultra-thin broadband metamaterial absorber. Appl. Phys. A.

[39] Cencillo-Abad P, Ou JY, Plum E, Zheludev NI (2017). Electro-mechanical light modulator based on controlling the interaction of light with a metasurface. Sci. Rep..

[40] Gholipour B, Zhang JF, MacDonald KF, Hewak DW, Zheludev NI (2013). An all-optical, non-volatile, bidirectional, phase-change meta-switch. Adv. Mater..

[41] Zheludev NI, Plum E (2016). Reconfigurable nanomechanical photonic metamaterials. Nat. Nanotechnol..

[42] Cencillo-Abad P, Ou JY, Plum E, Valente J, Zheludev NI (2016). Random access actuation of nanowire grid metamaterial. Nanotechnology.

